# Evaluation, Validation, and Recognition of the Point-of-Care Circulating Cathodic Antigen, Urine-Based Assay for Mapping *Schistosoma mansoni* Infections

**DOI:** 10.4269/ajtmh.19-0788

**Published:** 2020-05-12

**Authors:** Daniel G. Colley, Charles H. King, Nupur Kittur, Reda M. R. Ramzy, William Evan Secor, Merlene Fredericks-James, Giuseppina Ortu, Michelle N. Clements, Eugene Ruberanziza, Irenee Umulisa, Udo Wittmann, Carl H. Campbell

**Affiliations:** 1 Schistosomiasis Consortium for Operational Research and Evaluation (SCORE), Center for Tropical and Emerging Global Diseases, University of Georgia, Athens, Georgia;; 2 Department of Microbiology, University of Georgia, Athens, Georgia;; 3 Center for Global Health and Diseases, Case Western Reserve University, Cleveland, Ohio;; 4 National Nutrition Institute, General Organization for Teaching Hospitals and Institutes, Cairo, Egypt;; 5 Division of Parasitic Diseases and Malaria, Centers for Disease Control and Prevention, Atlanta, Georgia;; 6 Ministry of Health and Wellness, Castries, St. Lucia;; 7 Santé Publique France, Paris, France;; 8 Schistosomiasis Control Initiative, London, United Kingdom;; 9 Medical Research Council, Clinical Research Trials Unit, University College London, London, United Kingdom;; 10 Malaria and Other Parasitic Diseases Division, Neglected Tropical Diseases and Other Parasitic Diseases Unit, Rwanda Biomedical Center, Ministry of Health, Kigali, Rwanda;; 11 African Leaders Malaria Alliance, Dar-es-Salam, Tanzania;; 12 Consult AG Statistical Services, Zurich, Switzerland

## Abstract

Efforts to control *Schistosoma mansoni* infection depend on the ability of programs to effectively detect and quantify infection levels and adjust programmatic approaches based on these levels and program goals. One of the three major objectives of the Schistosomiasis Consortium for Operational Research and Evaluation (SCORE) has been to develop and/or evaluate tools that would assist Neglected Tropical Disease program managers in accomplishing this fundamental task. The advent of a widely available point-of-care (POC) assay to detect schistosome circulating cathodic antigen (CCA) in urine with a rapid diagnostic test (the POC-CCA) in 2008 led SCORE and others to conduct multiple evaluations of this assay, comparing it with the Kato–Katz (KK) stool microscopy assay—the standard used for more than 45 years. This article describes multiple SCORE-funded studies comparing the POC-CCA and KK assays, the pros and cons of these assays, the use of the POC-CCA assay for mapping of *S. mansoni* infections in areas across the spectrum of prevalence levels, and the validation and recognition that the POC-CCA, although not infallible, is a highly useful tool to detect low-intensity infections in low-to-moderate prevalence areas. Such an assay is critical, as control programs succeed in driving down prevalence and intensity and seek to either maintain control or move to elimination of transmission of *S. mansoni*.

## INTRODUCTION

The microscopy-based Kato–Katz (KK) thick smear fecal assay is highly specific and has been used widely for more than 45 years^1^ to determine the prevalence and gauge the intensity of *Schistosoma mansoni* infections. Where prevalence levels are high, it performs well, although it requires stool collections and qualified technical personnel to prepare and read slides. However, in low-prevalence areas or after successful control interventions, because of its lack of sensitivity, the KK assay underestimates true local prevalence of infection in such settings.^2,3^ As prevalence has decreased in many places because of preventive chemotherapy with praziquantel (PZQ), it has become increasingly apparent that Neglected Tropical Disease (NTD) program managers need a more sensitive and more field-applicable assay for mapping *S. mansoni* infection than stool microscopy.

In December 2008, the Bill & Melinda Gates Foundation (BMGF) funded the Schistosomiasis Consortium for Operational Research and Evaluation (SCORE; https://score. uga.edu) to conduct operational research on the control and elimination of *S. mansoni* and *Schistosoma haematobium.* One objective of this program was to develop and evaluate mapping and diagnostic tools that would help NTD program managers in their efforts to control schistosomiasis. Even earlier in 2008, while the BMGF was still considering the SCORE proposal, Dan Colley, principal investigator of SCORE, and BMGF officers Julie Jacobson and Debbie Burgess met regarding the pending commercialization and availability of a point-of-care (POC) cassette assay that could detect circulating cathodic antigen (CCA) from *S. mansoni* in urine specimens. Circulating cathodic antigen is a genus-specific glycan antigen discovered in the mid-1970s that is vomited into the blood by adult schistosome worms living in blood vessels and is excreted in the urine of the mammalian host.^4^ An earlier rapid diagnostic test detecting CCA appeared to have great promise as a mapping tool for *S. mansoni* infection,^5^ but that assay was not widely commercialized for programmatic use and was later unavailable. Subsequently, a strong case was made for using rapid diagnostic tests for schistosomiasis mapping.^6^ In 2008/2009, the POC-CCA assay became widely available, but had not been widely used, especially in settings of different prevalence levels or in side-by-side comparisons with KK stool microscopy. Also, when such comparisons had been performed, the KK assay was almost always treated as a “gold standard,” despite the logical inappropriateness of doing so, given its low sensitivity at lower levels of prevalence.

Propitiously, sales of the POC-CCA assay by Rapid Medical Diagnostics (RMD; Pretoria, South Africa) began in September 2008. Further evaluation of the POC-CCA test was included in the SCORE proposal to the BMGF because of the potential for this assay to be an important tool for mapping *S. mansoni* infections.

This article describes the various studies and evaluations conducted by SCORE to assess field performance of the POC-CCA assay. The evaluations included the SCORE five-country study, conducted in 2010; several focused studies conducted in Kenya; a systematic review using publications up to June 2015 of the KK-POC-CCA relationship; countrywide mapping studies in places with low and very low or no prevalence; and considerations regarding trace readings. It also describes remaining issues related to quantitation of infection intensity and POC-CCA batch variability, as well as SCORE’s contributions to guidance and guidelines by the World Health Organization (WHO). Note that SCORE has had continued interest in the POC-CCA assay, but it has never had any financial link to the manufacturer or sellers of the commercial version of the POC-CCA test.

## FIRST EVALUATIONS OF THE POC-CCA ASSAY FOR MAPPING OF *S. MANSONI*: THE FIVE-COUNTRY STUDY

To better understand POC-CCA performance, in September 2009, SCORE developed a request for applications (RFA) with input from its Advisory Committee and in consultation with WHO/NTD and WHO/AFRO. The RFA requested proposals to compare the POC-CCA assay with the KK assay performed on one, two, or three stools, with two slides per stool, for the detection of *S. mansoni* eggs from schoolchildren. These parallel evaluations were to be carried out in sites thought to have low (10–24%) or moderate prevalence (25–50%) for *S. mansoni* infections, or areas thought to have mixed *S. mansoni* and *S. haematobium* infections with the prevalence of each species estimated to be ≥ 25%. The primary question was, “Is the POC-CCA assay just as good as the KK?” The primary outcome was to be the non-inferiority comparison of a single urine POC-CCA assay with the KK assay results from the two slides from the first of the three sequentially collected stools. This comparator was chosen to simulate the single stool sample KK testing used by most control programs, rather than the standard research use of three stool specimens. The results comparing a POC-CCA single urine assay with the KK assay data from all three collected stools were also analyzed, so as to evaluate the POC-CCA against KK assessments that would have increased sensitivity.

SCORE shared the RFA with 16 groups of investigators and received 13 proposals, of which five were funded. The five funded projects were based in Cameroon, Côte d’Ivoire, Ethiopia, Kenya, and Uganda. The sites in both Côte d’Ivoire and Cameroon had some level of mixed infections with *S. haematobium*, although the predominant species was *S. mansoni*.

Each of the five programs that were part of this project analyzed and subsequently published their own data.^7–11^ In some studies, both the prevalence and the intensity of *S. mansoni* infections were assessed. Eggs per gram (EPG) of feces reflected the intensity of infection for KK. Relative intensity of infection was scored with the POC-CCA by comparing the test band with the control band to give a score of trace (considered as a weak positive), 1+, 2+, or 3+.^11^ The SCORE secretariat undertook the analyses and publication of the overall combined data with the other Five-Country Study consortium members.^12^

Point-of-care-CCA diagnostic accuracy was further investigated by stool-based real-time polymerase chain reaction analyses on 905 stools selected from among the five-country study specimens, with oversampling of specimens from study participants who had discordant POC-CCA and KK results. This additional assay on some of the specimens provided another data set for the subsequent latent class analyses (LCAs) of POC-CCA diagnostic accuracy.^12^ In addition, the project in Ethiopia included data from an area of Ethiopia without schistosomiasis to determine whether the POC-CCA assay would yield false-positive results in a non-endemic area. POC-CCA tests of 100 children in this area were all negative, except for one trace result. KK assays on stools of these children were uniformly negative for *S. mansoni*, but 50% of them were positive for at least one soil-transmitted helminth.^12^

Data from all 4,405 children with parallel KK and POC-CCA results demonstrated that the POC-CCA urine assay was just as good as the KK assay for mapping *S. mansoni* infections and better in many circumstances. The urine specimens were easier to collect than stool, and the assay was much simpler to perform. Based on LCA, the POC-CCA was more sensitive (86% versus 62%) than KK but less specific (72% versus ∼100%) than duplicate KK smears from one stool. The sensitivity of the POC-CCA was also much better than the KK assay for infection intensities of < 100 epg. The relationship between POC-CCA and KK assays varied by prevalence: prevalence of 50% by KK corresponded with the prevalence by POC-CCA of 72%, whereas a 10% KK prevalence was roughly equivalent to a prevalence of 46% by POC-CCA. Subsequent studies by SCORE and analyses by others have further characterized this nonlinear relationship.^13,14^

One concern had been that the POC-CCA would be too expensive for program use because it was a commercial product. At that time, the price per cassette was higher than that of the materials used to perform the KK. However, in another SCORE study, once all of the expenditures were considered, including the additional personnel time and return field visits required by KK, the two tests were found to be comparable in cost.^15^ By the time of this writing in 2019, the cost of the POC-CCA assay has decreased even further because of its more widespread use and purchases in bulk.

An unfortunate aspect of this five-country study was that along with the standard commercial POC-CCA assay kits supplied by the manufacturer (RMD) for the studies, RMD included an “experimental/lower sensitivity” POC-CCA assay thought by RMD to be an improvement over the standard assay. In reality, this “experimental” POC-CCA assay was decidedly inferior to the standard version for detecting low-intensity infections. The results using this substandard assay were intended to be analyzed in house and were not expected to be published. However, in several cases, results from this inferior/noncommercial version of the assay were published in the site-specific publications,^7,8,10,11^ which resulted in continued confusion about the overall performance of the POC-CAA.

### Kenyan studies on variability of POC-CCA results.

Although the five-country study demonstrated the potential utility of the POC-CCA assay for mapping of *S. mansoni* infections, many people, including those at the WHO making recommendations and guidelines for schistosomiasis control, continued to raise questions about its performance. Therefore, SCORE undertook several additional studies in Kenya related to POC-CCA assay performance to expand the database and further establish the rationale for its use to map *S. mansoni* infections. These assessments found no significant variability among the batches of the assays that they studied at that time, and that intra- and inter-reader variability was insignificant. Although there was day-to-day variability in POC-CCA readings (18% of urines from the same individuals tested on multiple days), it was much less than day-to-day variability of the stool KK assays (48% of the individuals).^16^ In children who were POC-CCA positive but KK negative based on three stools/two slides each, 47% became POC-CCA negative after treatment with a single dose of praziquantel (PZQ); 34% of those remaining POC-CCA positive after one treatment became negative after a second PZQ treatment.^16^

### Systematic review of the relationship between KK and POC-CCA.

SCORE developed additional insight into the nonlinear relationship between KK and POC-CCA prevalence through a systematic review of all 19 published articles as of June 2015 that directly compared these assays.^13^ At a prevalence greater than 50% by KK, the two assays yielded approximately the same prevalence in terms of programmatic considerations, although the POC-CCA prevalence was often somewhat higher. By contrast, based on 21 data sets from 11 applicable studies, when KK prevalence was < 50%, the prevalence by POC-CCA was between 1.5-fold higher (at a higher prevalence) and 6-fold higher (at the lower prevalence). This POC-CCA-to-KK–positive result ratio increased as KK prevalence decreased.^13^ Five of the publications compared the intensity of infection by KK epg with the visual band density using the POC-CCA assay. There was a clear trend, with those with darker POC-CCA band readings having higher median stool epg than those with lower density visual bands.^13^ In addition, a recent publication has provided a scoring system to aid in visual grading of the intensity of the reaction band observed.^17^

## SCORE-SUPPORTED MAPPING STUDIES COMPARING THE POC-CCA ASSAY WITH THE KK ASSAY FOR MAPPING IN LOW TO VERY LOW PREVALENCE AREAS

In addition to the five-country study, SCORE has supported several other comparisons of the KK assay and the POC-CCA assay in multiple countries, especially those considered to have low or no prevalence of *S. mansoni*. These include studies in Ecuador (non-endemic), Burundi, and Rwanda (prevalence based on sentinel site surveillance before remapping < 2% by KK), St. Lucia (considered no longer endemic), Egypt (area-wide prevalence in the Nile delta < 2% by KK), and the previously mentioned 100 children in an area of Ethiopia that was non-endemic for schistosomiasis.

### Prevalence mapping studies in Burundi and Rwanda.

The mapping studies in Burundi and Rwanda were conducted to inform studies being planned on the elimination of *S. mansoni* in these countries. Burundi and Rwanda had had consistent annual mass drug administration (MDA) programs for 6 or 7 years.^18–20^ The MDA implementation differed somewhat between the two countries, but in both, annual MDA of PZQ was performed in those areas determined to need it, and based on KK sentinel site monitoring, both countries had achieved < 2% prevalence. The SCORE mapping studies, which followed these MDA programs, were conducted collaboratively with the respective Ministries of Health (MOHs), the Schistosomiasis Control Initiative (SCI), and the END Fund and were originally designed to estimate nationwide *S. mansoni* prevalence by POC-CCA testing of a single urine specimen from each of 50 13- to 14-year-old children per school in each of 400 schools. In approximately half of those schools, the POC-CCA assays were also compared with KK stool testing results (one stool/two slides) from the same children who provided urines. In Burundi, prevalence by POC-CCA was 42.8% in the 17,331 children tested.^19^ Of these, 8,482 children were also tested using KK, yielding a prevalence of 1.5% by the KK assay and 41.3% by the POC-CCA assay. In Rwanda, testing similar numbers of children in the same ways yielded remarkably similar KK and POC-CCA data and analyses, with POC-CCA results indicating *S. mansoni* infections, albeit most of low intensity, in all 31 mapping units.^20^

A subset of urine specimens (398) from Burundi was selected from eight sentinel site schools to include a spectrum of prevalence levels, spanning 0–20% by the KK assay and 12–90% by the POC-CCA assay. These urine samples were sent to Leiden University Medical Center (LUMC) to be further tested by the very sensitive and schistosome-specific up-converting phosphor lateral flow circulating anodic antigen (UCP-LF CAA) assay.^21^ In this selected subset, the average prevalence levels by KK, POC-CCA, and UCP-LF CAA were 6.8%, 53.5%, and 46.5%, respectively. Sixty-one percent of the positive POC-CCA readings were traces. Further analysis by LCA indicated that the POC-CCA assay outperformed the KK assay at the low infection intensities in Burundi. Latent class analysis estimated that approximately 50% of trace readings were true positives.^22^ Furthermore, it was estimated that the KK assay missed ∼85% of infections, albeit most of those were likely of light intensities or egg negative. Again, the Rwanda data and LCA analyses provide conclusions very similar to those from the Burundi mapping.^20^

It is clear based on the mapping studies in Burundi and Rwanda that even when prevalence is very low by KK, it is much higher by POC-CCA. It is also clear that most of those who are KK negative and POC-CCA positive have trace readings. SCORE has referred to the many individuals in low-to-moderate prevalence areas with positive POC-CCA results and no eggs found in stools (at least by the KK assay on two slides from one stool) as “egg-negative/worm-positive” (see in the following text).

### Mapping study in St. Lucia.

At one time, the Caribbean island nation of St. Lucia was highly endemic for *S. mansoni*. However, following the Research and Control Project on schistosomiasis (1967–1981)^23^ and during considerable economic development in the intervening 40 years to the present, schistosomiasis seems to have disappeared. In collaboration with the Ministry of Health and Wellness of the Government of St. Lucia, the Pan American Health Organization, and the Centers for Disease Control and Prevention (CDC), SCORE assisted in mapping study of all primary schools (*n* = 63) on St. Lucia.^24^ This mapping included 16% of the 8,985 children aged between 8 and 11 years on the island and included collection of urine (*n* = 1487) and finger-stick blood (*n* = 1455) samples. Fourteen percent (*n* = 209) of the children providing a urine sample had a trace (*n* = 150) or 1+ (*n* = 59) POC-CCA result in the field. Some of the samples were also reassessed as trace or 1+ readings when retested by POC-CCA at the University of Georgia. However, on subsequent testing of suspected positive urines by the UCP-LF CCA assay at LUMC, although there were a few, very low, inconsistently positive results on multiple UCP-LF CAA tests, they were not from the same urines that had low positive POC-CCA values. Similarly, although there were some children (*n* = 8; 0.6%) with initial anti-schistosome–soluble egg antigen ELISA antibody results slightly higher than the cutoff, none tested positive for schistosome infection by confirmatory western blot using the *S. mansoni* adult microsomal antigen.^25^ Furthermore, there was no correlation among the children who tested positive by POC-CCA, those who tested positive by the UCP-LF CAA, or those who were initially positive by anti-SEA ELISA. The UCP-LF CAA assay and the western blot assay are considered by many as confirmatory assays. Therefore, we concluded that negative and inconclusive results using those two assays meant that none of the children in the study was confirmed to have *S. mansoni* infection.^24^

## INTERPRETING TRACE-POSITIVE READINGS OF THE POC-CCA ASSAY

It is clear based on the data from very low-prevalence settings that an important challenge is how to read and interpret visually faint bands, called “trace.” The manufacturer’s instructions stated that trace should be considered as positive, and, eventually, most of the evidence generated by LCAs and other studies led the WHO to also state that trace results should be considered positive. This appears to be appropriate, except in areas with extremely low prevalence—around 1–2% by KK. Although not every trace reading is a true positive, when the POC-CCA is used for mapping purposes to determine MDA interventions, somewhat overestimating prevalence by scoring trace results as positive can ensure that areas with infected individuals are not left untreated.

SCORE made attempts to use smartphone and tablet apps as quantitative readers to overcome the challenge of both the trace readings and the subjective interpretation of the intensity of the “test line” compared with the intensity of the “control” band. Although the applications achieved the goal of providing quantitative readings of POC-CCA band intensity, the goal of distinguishing between trace results that are false positive versus true positive has thus far not been achieved.

### How can someone be KK egg–negative and POC-CCA–positive?

There are multiple possible explanations for the discrepancy sometimes observed between a negative KK and a positive POC-CCA result, especially when the POC-CCA readings are trace or 1+. One is that the two assays measure different schistosome life stages. The KK measures only eggs that are excreted in the feces, whereas the POC-CCA detects a product from living adult worms excreted in the urine. Furthermore, the relationship between the number of worms and the number of eggs excreted at any given time point during this yearslong infection is not known. In fact, it is likely that the relationship between eggs and adult worms (and thus CCA production) changes over time during this chronic infection.^26^ When both assays are used appropriately by trained users, some of the possible reasons for egg-negative CCA-positive schistosomiasis are as follows:

1. The KK assay is insensitive and missed an egg.
 a) The egg was in another part of the stool.
 b) The egg was excreted on a different day.
2. The POC-CCA result was a false positive.
3.The POC-CCA readers/technicians were insufficiently trained or trained differently in different programs/teams and read a negative result as a positive.
4. The person harbors a bisexual infection, but the female worms became infertile.
5. The person harbors a bisexual infection, but anti-fecundity immunity reduced or stopped egg production.
6. The person harbors a single sex infection.

What should an NTD program manager do when confronted with a person who is egg negative/POC-CCA positive, particularly when there are few individuals with POC-CCA readings greater than 1+ and an overall prevalence by the KK assay that is very low? This issue arose in the Burundi^19^ and Rwanda studies,^20^ where prevalence in some villages was very low or zero by KK, but many children were POC-CCA positive, albeit with a preponderance of trace readings (see in the following text). If these children had worms that were excreting eggs, albeit at low levels, they still could be at risk for morbidity or pose a risk for transmission.

Regardless of the reason for someone being egg negative/POC-CCA positive, understanding the answer to the question “If the worms are not making eggs, are they causing morbidity?” has important implications for control of morbidity and elimination activities.

### Studies of POC-CCA positives in areas of very low prevalence in Egypt.

SCORE collaborated with the Ministry of Health and Population of the government of Egypt to conduct an intensive evaluation of egg excretion from children in an area of very low schistosomiasis prevalence by KK (< 2%). These children lived in three districts that had been under schistosomiasis control for many decades.^27,28^ The three districts chosen for the SCORE studies of egg excretion had prevalence levels of 1.2%, 0.0%, and 0.9% by the KK assay based on more than 2,000 children tested in a mapping study in 2016.^27^

In late 2017, the study enrolled 45 children who had POC-CCA results of trace or 1+ but who were KK negative on initial screening. The primary study question was whether such egg-negative/trace or 1+ POC-CCA–positive children in this area of very low prevalence excrete detectable *S. mansoni* eggs over a 30-day period. Stool and urine samples were collected every day from each child for 30 days. Stool samples were examined by the KK assay (one stool/four slides), and all *S. mansoni* egg-negative stools were further tested by the miracidia hatching test (MHT). Daily urine specimens were examined by one POC-CCA assay. The data clearly indicated those KK egg-negative children with trace or 1+ POC-CCA readings very infrequently (one of 1,388 stools; 0.1%) pass *S. mansoni* eggs.^29^ Thus, such children are unlikely to have ongoing egg-focused morbidity or contribute to the transmission of schistosomiasis.

To evaluate whether these children harbored low, undetectable numbers of adult worms or the POC-CCA results in this setting were false positives, SCORE’s Egyptian collaborators investigated whether the trace or 1+ POC-CCA readings of KK egg-negative children would change to negative POC-CCA following one, two, or three treatments with PZQ. Of the 45 children in the 30-day study of stools and urine, 44 participated in this follow-up treatment study.^30^ The first and second PZQ treatments were conducted 3 months apart, and 5 weeks separated the second and third PZQ treatments. Stool and urine specimens were collected 3 months following the initial PZQ treatment, 3 weeks following the second PZQ treatment, and then 3 weeks after the third PZQ treatment. For each evaluation, stool and urine specimens were collected on three successive days. Stool specimens were examined by the KK assay (one stool/four slides), and all *S. mansoni* egg-negative stools were further tested by the MHT. Each urine sample was examined by one POC-CCA. Over the study period, all stool samples from study subjects remained *S. mansoni* egg-negative by KK and MHT. Of the POC-CCA test results on the first 3 days of urine collections 3 months following the initial treatment, 29.6% were negative, 61.4% had trace-positive POC-CCA results, and 9.1% had POC-CCA 1+ results. Following the two additional PZQ treatments, the POC-CCA test results fluctuated between negative, trace, and 1+, but did not consistently become negative. Furthermore, there were no differences between the proportions of POC-CCA trace and 1+ results obtained in the first day (70.5%) and on the last day of the study (72.7%). The lack of consistent change in test results to negative after multiple treatments makes it likely that the trace and 1+ POC-CCA readings in this very low prevalence area that had received control interventions for decades were false positives.^30^ We conclude that these children are neither at risk for schistosomiasis-related morbidity nor do they represent a public health problem in terms of contributing to the transmission of schistosomiasis.

### The challenges of interpreting trace POC-CCA readings in different places.

Based on the LCA studies of the data from the SCORE mapping in Burundi and Rwanda, approximately 50%^20,22^ or at least 50% of the trace POC-CCA readings were estimated to be true positives.^20–22^ In mapping settings like these, where MDA had been going on for only 6 or 7 years and prevalence is low by the KK assay but high by POC-CCA, we propose categorizing trace results as positive. This would ensure treatment is provided to people in areas that would benefit by treatment but that would be excluded based on KK mapping. However, in areas that are nearing or perhaps have achieved elimination, for example, in St. Lucia and the areas in Egypt where the aforementioned studies were conducted and control interventions have been going on for many decades, it appears that trace POC-CCA readings are very likely to all be false positives.^24,29,30^

In deciding whether POC-CCA trace readings should be interpreted as false or true positives, we propose that both the control history and current prevalence of the location should be considered. As indicated earlier, the three villages in the Egyptian studies had prevalence levels of 1.2%, 0.0%, and 0.9% by the KK assay based on more than 2,000 children tested. In the same survey, the POC-CCA prevalence in these villages, testing the same children, was 9.8%, 10.8%, and 7.6%, respectively,^27^ and almost 90% of those read as positive by POC-CCA were trace or 1+ readings. Furthermore, the mapping in St. Lucia^24^ was performed more than three decades after extensive interventions were applied across the country, and St. Lucia had undergone widespread development with much of the country moving from an agricultural-based to a tourism-based economy.^31,32^ It seems likely that any schistosomiasis transmission dynamics encountered in St. Lucia and the three study villages in Egypt would differ considerably from those taking place in Burundi and Rwanda, each of which had undergone selective area MDA with PZQ for only 6 years at the time of the mapping surveys.^18,33,34^ Another difference in the two different types of settings (Table 1) is the proportion of children with trace POC-CCA readings (Egypt = 8.4%; St. Lucia = 10% compared with Burundi = 27%; Rwanda = 30%).

**Table 1:** Comparison of proportion of children with trace POC-CCA readings in four different settings

		Egypt	St. Lucia	Rwanda	Burundi
Current data Sampling		465 schoolchildren in three districts surveyed with both KK and POC-CCA	1,487 children from island-wide mapping with POC-CCA	8,697 children from country-wide mapping who have data on both KK and POC-CCA	9,371 children from country-wide mapping who have data on both KK and POC-CCA
Source of data		Haggag et al.^28^	Mapping dataset from St. Lucia^24^	Mapping dataset from Rwanda^20^	Ortu et al.^19^
KK positive		3 (0.6%)	Not tested	172 (2.0%)	157 (1.7%)
POC-CCA result	Negative	364 (78%)	1,278 (86%)	5,438 (62.5%)	5,508 (58.8%)
	Trace	39 (8.4%)	150 (10%)	2,513 (28.9%)	2,827 (30.2%)
	1+	48 (10.3%)	59 (4%)*	479 (5.5%)	648 (6.9%)
	2+	11 (2.4%)		147 (1.7%)	204 (2.2%)
	3+	3 (0.6%)		120 (1.4%)	184 (2.0%)
Treatment history in the area					
		Schistosomiasis control for many decades	Three decades of extensive interventions, and development of a tourism-based economy	Selective praziquantel mass drug administration for 6 years before mapping survey	Selective praziquantel mass drug administration for 6 years before mapping survey

KK = Kato–Katz; POC-CCA = point-of-care circulating cathodic antigen assay.  ^*^ = 1+, 2+ and 3+ readings.

We hypothesize that in areas where transmission is clearly continuing, albeit perhaps at relatively low levels in most areas, such as in Burundi and Rwanda, there are many more low-level infections that are not detected by the KK assay but are detected by trace and 1+ POC-CCA readings, and at least 50% of these are true positives. This hypothesis could be tested in Burundi, Rwanda, or other similar locations by evaluating the clearance (or lack thereof) of CCA following PZQ treatments.^16,30^ By contrast, in locations where multiple control interventions and/or development have been going on for decades, a child who is KK negative and POC-CCA trace or 1+ reading, in the face of either extremely low or no transmission, is almost certainly false positive, as described earlier with the studies in Egypt and St. Lucia. Any test that has less than 100% specificity will begin to have larger percentages of false-positives as the true infection level goes down. Therefore, we propose that if the prevalence by POC-CCA in a given area goes below a given threshold, perhaps 8% or 10%, and all readings are trace or 1+, and there is no other evidence of schistosome transmission (either by KK, MHT, or xenomonitoring of snails), the POC-CCA readings are most likely false positives. At that point, confirmatory assays and surveillance schemes will be needed to monitor for sporadic, resurgent transmission.

## ADDITIONAL CONSIDERATIONS IN USING POC-CCA FOR MAPPING

### The lack of quantification provided by the POC-CCA assay.

The visually observed band density of the POC-CCA only provides a semi-quantitative correlate with the egg count–based intensity reading that can be provided by KK. Nevertheless, there is a reasonable relationship between the KK EPG estimate and the intensity of the POC-CCA band,^13^ which can allow for a general estimate of the intensity of infection. Furthermore, given the variability of the quantitative egg count obtained by the KK assay at low-to-moderate levels of intensity,^35^ this may not be as critical a difference as perceived by some.

### Variability in POC-CCA batches.

As noted earlier, there were no significant differences among the batches of POC-CCA assays evaluated in the studies by Mwinzi et al.^16^ However, this has not held true for some batches produced later by RMD and ICT Diagnostics (ICT; Noordhoek, South Africa) and sold to programs and researchers, for example, the “experimental/lower sensitivity” version of the test sent to investigators in the five-country study. In 2016, the company undertook “optimization” efforts to try to reduce the percentages of trace results. SCORE was involved in evaluating two optimization batches, referred to as Version 1 and Version 2. These evaluations were conducted on stored urine from three countries and on fresh urine specimens collected in Mwanza Region, Tanzania, in August and December 2016. The overall results from both optimized versions showed that, although specificity was higher, sensitivity was much lower than that of previous standard batches, and SCORE strongly recommended that these so-called by RMD optimized versions 1 and 2 not be distributed for mapping or study purposes. Nevertheless, RMD moved ahead and produced batches with less sensitivity, which they sold from December 2016 through March 2017. RMD and ICT failed to communicate this change in sensitivity to those who purchased their products during that period, compounding the confusion by those who used them. Thus, there are data from some studies that may not be useful nor comparable to most of the other studies conducted with the standard batches both in the same countries over time as well as between countries.

In October 2017, RMD made another change in the assay from requiring one drop of urine and one drop of buffer to requiring two drops of urine and no buffer. This change was announced to customers, and this is the version being sold to this day (spring 2019). This change was noted on the company’s website and did not alter the specificity or sensitivity of the standard POC-CCA cassette assay. In parallel urine testing, the sensitivity of this two drops of urine/no buffer version of the POC-CCA assay is similar to that of the earlier (pre-December 2016) standard version. As a continued precaution, SCORE recommends that the batch numbers and expiration dates be recorded for all mapping and research purposes and reported with any results.

Aberrant results have also been reported related to the use of the POC-CCA assay in Brazil.^36,37^ Some of these results may be attributable to the fact that Brazil requires the assay to be assembled in Brazil using components from the South African supplier, which could result in different levels of quality control of the final product,^37^ or this could be related to the issue of different batches of components mentioned earlier.

### *Schistosoma haematobium* and the POC-CCA.

Unfortunately, the POC-CCA assay is inconsistent for the detection of infection with *S. haematobium.*^38–41^ Thus, because the standard urine filtration assay for *S. haematobium* eggs is, like the KK, insensitive at low-to-moderate levels of infection intensity,^42,43^ a tool is still needed for mapping *S. haematobium* in low prevalence areas.

## SCORE’S CONTRIBUTIONS TO WHO GUIDANCE AND RECOMMENDATIONS RELATED TO POC-CCA USE FOR MAPPING OF *S. MANSONI* INFECTIONS

By the time the five-country study was published in early 2013,^12^ the individual country data and the combined data had been presented many times in many international venues, and findings that the POC-CCA was more sensitive and easier to do than the KK assay were widely known. At the SCORE Annual Meeting in May 2012, which included the SCORE Advisory Committee and two WHO/NTD representatives, SCORE was encouraged to draft a recommendation to the WHO/NTD office regarding the potential use of the POC-CCA. Subsequently, a draft statement was prepared and shared with all participants at the 2012 SCORE Annual Meeting for input. After multiple revisions, the final statement was sent to WHO/NTD on May 25, 2012. Providing data from the five-country study and noting that others had generated similar data, the final cover email and statement asked that the WHO take the following under advisement: “SCORE recommends that a single urine examination by the commercially available POC/CCA cassette-based test can be used—and in our estimation should be used—instead of a single stool examination by the KK method to assess the prevalence of *S. mansoni* infections in children of school age for the purposes of mapping *S. mansoni* for decision-making in regard to preventive chemotherapy.” They never received a response from the WHO. An additional 3 years passed before this issue was addressed by the WHO and the NTD-STAG Global Working Group on Monitoring and Evaluation of Preventive Chemotherapy approved the POC-CCA “for use in monitoring and evaluation of *S. mansoni* infection control and elimination programs.”

Recently, there has been a major modeling effort published regarding the relationship of the KK assay to the POC-CCA across the spectrum of prevalence levels.^14^ SCORE, SCI, and the WHO assisted the investigators in compiling all the existing comparative data on these two assays. The resulting article describes the relationship between KK prevalence and different levels of POC-CCA prevalence and states the implications of this relationship for applying current WHO guidelines, which are based on KK prevalence levels, to results using POC-CCA. Based on this comprehensive analysis, the WHO is currently considering a recommendation relating these two assays, leading to a recommendation that it is acceptable to use the POC-CCA to evaluate the prevalence of *S. mansoni*. Paired recommendations for cutoffs based on CCA or KK prevalence levels will allow NTD program managers to use either assay to estimate the prevalence that would be expected using the other assay. It is anticipated that some programs in areas that still have high prevalence levels may continue to use the KK, with which they are familiar, and which also provides an opportunity to assess soil-transmitted helminths. However, in low-to-moderate prevalence areas, the POC-CCA will clearly provide a better estimate of the actual prevalence of *S. mansoni*. Regardless of expected prevalence, the POC-CCA is an easier test to use because of the relative ease of collecting urine samples compared with stools and the faster time to obtaining results.

## OTHER SCORE EFFORTS RELATED TO DIAGNOSTIC TOOLS

SCORE has also supported research to help develop an assay for both for *S. mansoni* and *S. haematobium* that would be both highly specific and highly sensitive. SCORE-supported work focused on the UCP-LF CAA assay and its use as a confirmatory assay. These efforts are described elsewhere in this supplement.^44^ In addition, because of the extreme need, SCORE also supported initial efforts to use CAA in the development of a rapid diagnostic^44^ that would be suitable for mapping both *S. mansoni* and *S. haematobium.*

## FINAL THOUGHTS ON SCORE’s EFFORTS TO EVALUATE THE USEFULNESS OF THE POC-CCA ASSAY FOR MAPPING *S. MANSONI* INFECTIONS AND DEVELOPMENT OF FUTURE DIAGNOSTIC TOOLS

The widespread commercial availability of the POC-CCA assay for mapping of active *S. mansoni* infections coincided with the start of SCORE. This provided an excellent opportunity to evaluate this field-friendly assay and relate it to the standard diagnostic assay at the time, the KK. It should be reiterated that SCORE has never had any financial link to the manufacturers or distributors of the POC-CCA (RMD and ICT) and that all of the multiple SCORE-funded evaluations in all 12 countries involved in one study or another were performed solely to determine how it would perform as needed for mapping infection with *S. mansoni*.

SCOREs presentations and publications have consistently stated that considering the KK assay the gold standard in evaluations of specificity and sensitivity of the POC-CCA is inappropriate because of the known insensitivity of the KK assay, especially in areas with low-to-moderate prevalence. Nevertheless, some groups, including the Cochrane systematic reviews, continue to make such mistaken comparisons.^45^

Many groups have pursued multiple other diagnostic assays for detection of schistosome infections including using antibody assays that detect either current or past infections, nucleic acid amplification tests, as well as assays to detect other carbohydrates and proteins of schistosomes. Furthermore, SCORE has participated in at least five meetings and conference calls to develop suitable target product profiles (TPPs) for schistosomiasis diagnostics and has strongly advocated for the development needed to make these TPPs a reality for programs seeking to control and/or eliminate schistosomiasis. It is hoped that efforts will continue to develop and rigorously evaluate all such assays, and these efforts will lead to standardized, commercially available, field-applicable tools for mapping, elimination verification, surveillance, and individual diagnoses of schistosomiasis.
